# The merger that made us

**DOI:** 10.1186/s12915-020-00806-3

**Published:** 2020-06-24

**Authors:** Buzz Baum, David A. Baum

**Affiliations:** 1 MRC-Laboratory of Molecular Cell Biology and the Institute for the Physics of Living Systems, UCL, Gower Street, London, WC1E6BT UK; 2 University of Wisconsin – Madison, 430 Lincoln Drive, Madison, WI 53726 USA

Darwin was the first to imagine a tree of life with all living organisms at its branch tips, connected back through time to a single common ancestor at the base of the trunk. We now know that all known cellular life descended from a single rootstock, with bacteria and archaea, structurally simple prokaryotic cells that lack internal membrane-bound structures, making up the trees’ two main branches (see Fig. 1). Where on this tree, though, should we place animals and all the other organisms whose complex cells possess a nucleus and a labyrinthine endomembrane system, namely the eukaryotes? Phylogenetic analyses of eukaryotic genes have firmly established that eukaryotes first arose as the result of a merger of cells from two divergent, prokaryotic lineages [1, 2]. One of these two cells appears to have been a member of a subgroup of archaea, the so-called TACK archaea, which includes the widely studied *Sulfolobus*, whereas the other partner appears related to alpha-proteobacteria. Thus, the origin of eukaryotes is best depicted as a point of fusion on the tree of life (see large arrow on Fig. 1). Alpha-proteobacteria closely resemble mitochondria in many aspects of their structure and biochemistry, so these cells are thought to be the progenitors of mitochondria, leaving TACK as the presumed source of other components of the eukaryotic cell.

**Fig. 1:**
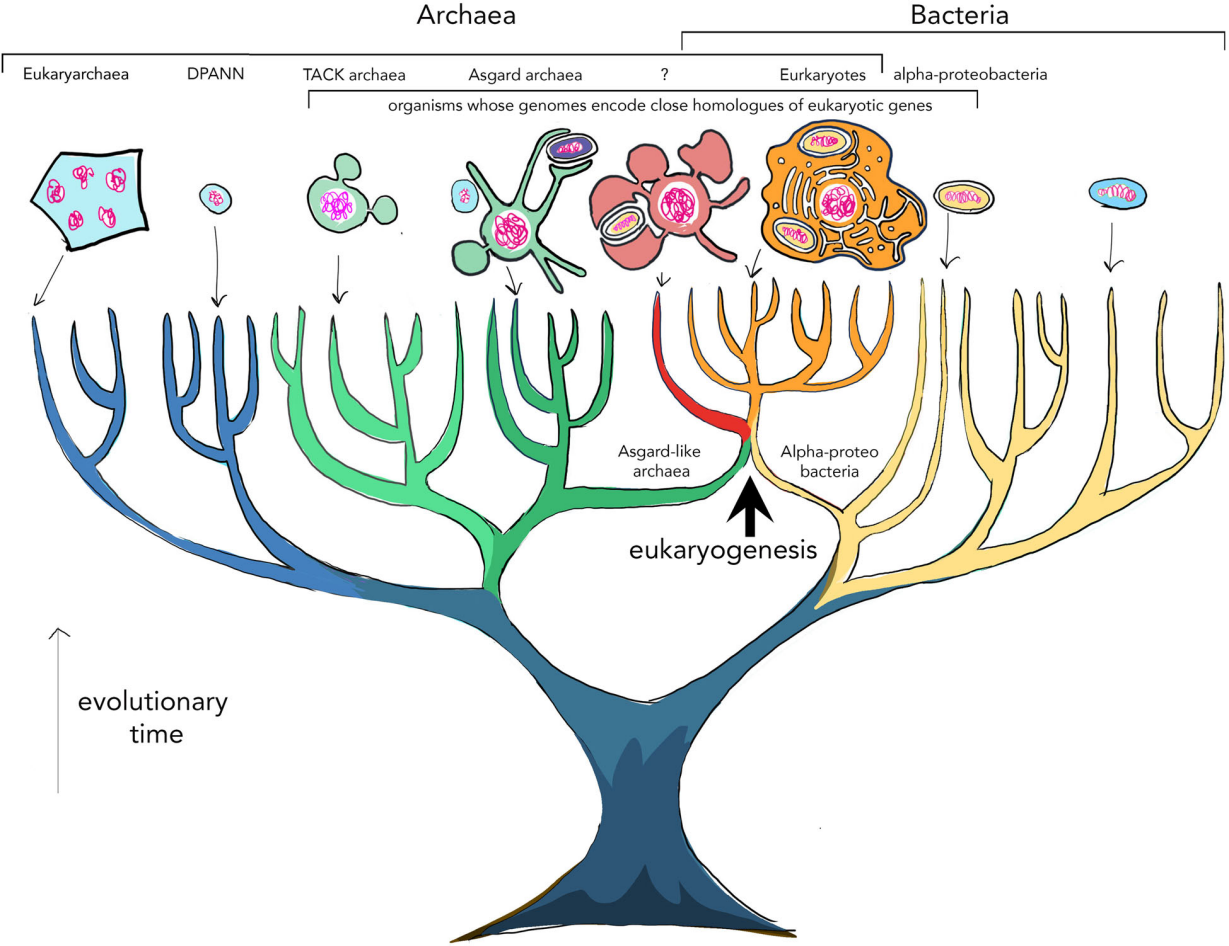
Tree of life: The tree summarizes the broadscale evolution of life on Earth. The two main branches depict the bacteria and archaea. Eukaryotes possess genes of both bacterial and archaeal ancestry and arose from the merger of a host cell, closely related to the Asgard superphylum of archaea, with a member of the alpha-proteobacteria, which gave rise to mitochondria. Drawings depict present-day examples of cells from different tips of the tree of life, emphasizing steps that likely connect the cell structures of TACK archaea, Asgard archaea, and eukaryotes. The question mark depicts a hypothetical intermediate along this path that is predicted by the inside-out model *(3)*, whose living descendants we would hope might be identified in the coming years. This is an Asgard-type archaeon that forms intimate connections with its obligate symbiotic partner—an alpha-proteobacterium

This raises a profound problem. How did two structurally simple prokaryotic cells come together to give rise to a complex eukaryotic cell? Historically, cell biological models assumed that the nucleus and endoplasmic reticulum evolved from the outside-in when invaginations of the plasma membrane of the archaeal host generated internal compartments via processes akin to endocytosis or phagocytosis. In 2014, however, we proposed an alternative “inside-out” model [3]. This flipped things around by envisioning the cytoplasm and endomembrane system gradually emerging from the elaboration of outward facing protrusions (see Fig. 2). Further, we speculated that the increase in the complexity of these protrusions over evolutionary time reflected a growing intimacy and increased metabolic exchange between the archaeal host and the once free-living proto-mitochondria. Whereas outside-in models assume that the plasma membrane remained in place during the evolution of eukaryotes, with the nuclear compartment arising from the coalescence of internalized membranes, the inside-out model posits that the inner nuclear membrane marks the boundary of the original archaeal host, with the endomembrane system and plasma membrane forming later as a result of the fusion of extracellular protrusions (see Fig. 2).

**Fig. 2:**
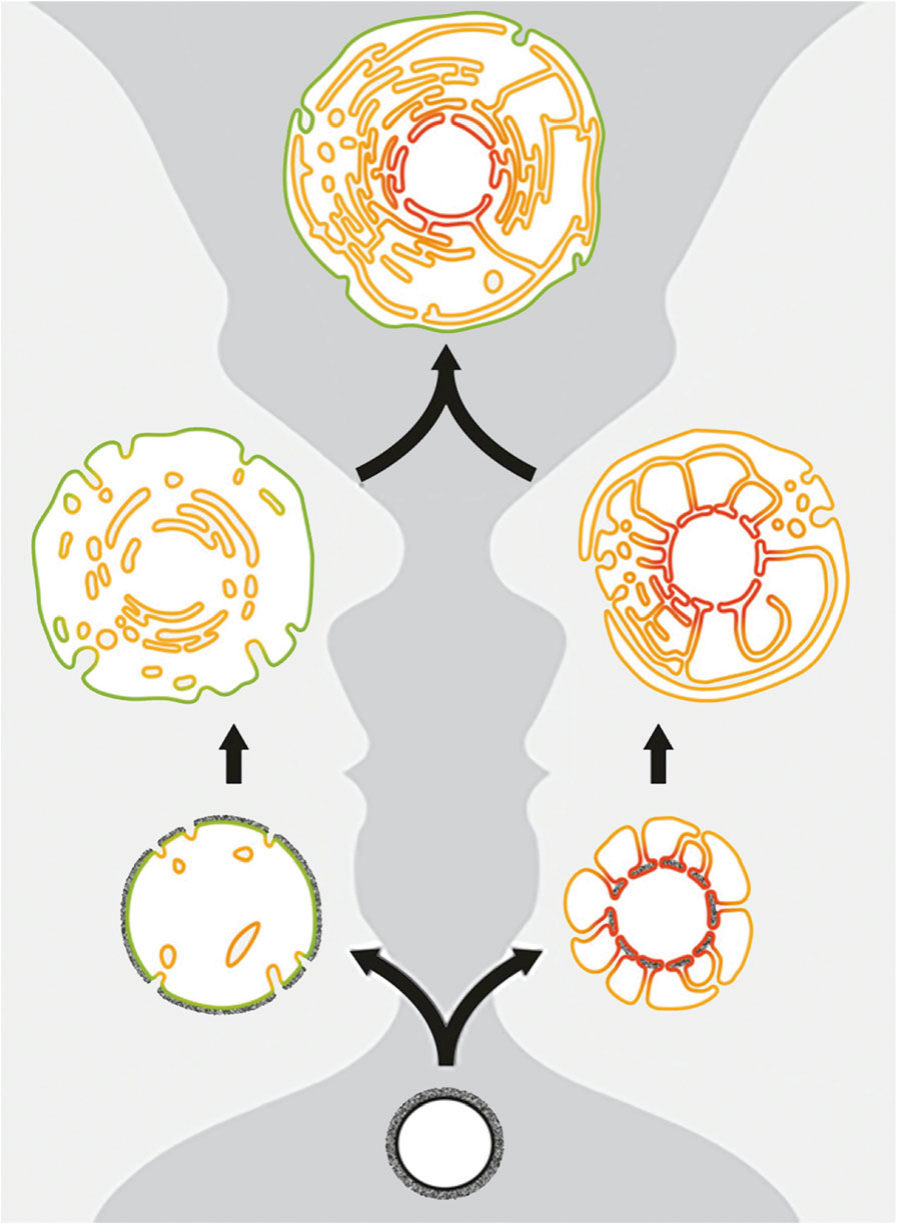
Two views of the evolution of the eukaryotic cell. The outside in model (left) posits that the archaeal and eukaryotic cell membranes (both shown in green) are homologous and that this membrane was invaginated to generate internal compartments that eventually fused to yield the endomembrane system and nuclear compartment. Inside-out model (right) suggests that the archaeal plasma membrane is homologous to the inner nuclear membrane (both shown in red) and that this membrane formed outward protrusions that eventually formed the outer nuclear membrane, endomembrane system, and cell membrane (modified from Baum D. *American J. Botany 2015,* 102: 1–12)

While one can imagine testing predictions of these alternative structural models of eukaryogenesis by studying the cell biology of present-day archaea, the large gulf that separates known TACK archaea and eukaryotes makes this difficult. With some interesting exceptions, TACK archaea have a standard prokaryotic organization with a membrane that consists of ether-linked, branched-chain lipids encased in a semi-crystalline glyco-protein coat. Progress has also been impeded by the fact that TACK archaeal cells tend to be small and to grow in environments we eukaryotes consider extreme—making them difficult to culture and study. Despite these challenges, work by several teams over a number of years has revealed a surprising number of cell biological features that TACK archaea share with eukaryotes. Like eukaryotes, TACK archaea have an ordered cell cycle, in which discrete phases of DNA replication and division are separated by gap phases [4]. In addition, many TACK archaea possess homologues of ESCRTIII proteins, which, like their eukaryotic counterparts, control cell division [5, 6], and also actin homologues that may help to shape these cells. Furthermore, TACK archaea share with eukaryotes a common pathway of N-linked glycosylation coupled transport of proteins across membranes, and they release membrane vesicles (including viral particles) generated via ESCRTIII-mediated scission [7], just as eukaryotes do. These latter facts imply that vesicle-based secretion in eukaryotes (including, multi-vesicular body formation) is based on pre-existing archaeal machinery. Nevertheless, many other proteins that play central roles in generating the dynamic and complex internal organization of a eukaryotic cell appear missing from the genomes of TACK archaea, including homologues of Dynamin, other ESCRT proteins, small regulatory GTPases and outer vesicle coat proteins, which play important roles in vesicle trafficking, and nuclear pore proteins. Without knowing the evolutionary origins of such proteins, it is difficult to reconstruct how cell structure acquired the levels of complexity seen in living eukaryotes.

Although a wide chasm remains, the gap between the prokaryotic and eukaryotic worlds closed significantly in 2015 when DNA isolated from environmental samples taken from the seabed off the coast of Norway was sequenced and organized into genomic assemblies [8]. This metagenomic work identified a “missing link”—a new record-holder for the closest living relative of eukaryotes. The Ettema group named this new microbe *Lokiarchaeum* and placed it close to the TACK clade as a new archaeal phylum, Lokiarchaeota. The genome of Loki, as it has come to be known, was found to contain a number of “eukaryotic signature proteins” not previously found in any prokaryote. This included homologues of the ubiquitin-ESCRT system, which controls membrane protein degradation and exosome formation in eukaryotes, and GTPases, including Rag-like GTPases, which in eukaryotes function at the lysosome. The genome also included actin homologues and, importantly, a small set of conserved eukaryotic-like actin regulators not found in TACK archaea. Strikingly, this was the case even though these organisms appear to possess classic archaeal lipids that are very different from those found in bacteria and eukaryotes. Since then, many new relatives of Lokiarchaeota have been identified, defining a new “Asgard” superphylum, some of whom may be even closer relatives of eukaryotes than Loki [2]*.* In the absence of live cultures, however, the morphology and behavior of Loki and other Asgards remained a matter of debate.

In 2020, a heroic 12-year effort by Imachi et al. to grow anaerobes from the bottom of the sea off the coast of Japan led to the fortuitous isolation of *Prometheoarchaeum syntrophicum*, another member of the Lokiarchaeota [9]. Although the team had hoped to generate pure cultures, this archaeon proved to be an obligate symbiont. Using electron microscopy, the team were able to obtain images of *P. syntrophicum* cells in mixed cultures. Strikingly, in these electronmicrographs many *P. syntrophicum* cells appeared similar to an early intermediate in the path to eukaryogenesis imagined in the inside-out model. Cells lacked internal membrane-bound compartments but, instead, possessed a central “nuclear-like” cell body, as well as external blebs and long finger-like protrusions. Further, in line with the inside-out hypothesis, Imachi et al. suggested that these extracellular protrusions may facilitate the exchange of material with their obligate extracellular symbionts.

The characterization of the first species of Lokiarchaeota has helped make the case that much of the machinery required to generate eukaryotic cellular complexity was already present in archaeal ancestors prior to the acquisition of mitochondria. However, it is clear that there are also many features of eukaryotic cell biology that are missing from Asgard archaea, including bacterial-type lipids, which are likely to be important for dynamic membrane fission-fusion reactions. Furthermore, there is no evidence of archaea undergoing a process akin to endocytosis, which frequently relies on the activity of Dynamin, a protein that eukaryotes appear to have acquired from the mitochondrial symbiont [10]. Thus, it seems clear that full-blown eukaryotic vesicle trafficking arose only after the archaeal eukaryotic ancestor established a close and stable association with a bacterial partner from which it acquired lipids and other, mainly metabolic, genes (see fusion point in Fig. 1). These bacterial novelties enabled proto-eukaryotic cells to elaborate on their basic cellular body plan—a likely pre-requisite for their subsequent radiation into important new ecological niches, from amoeboid-like predation to multicellularity.

The recent discovery of Loki and other Asgard archaea has ignited research into eukaryotic origins. At the same time, advances in metagenomics, phylogenetics, and archaeal molecular biology have put in place many of the tools required to test predictions of different models of eukaryogenesis. Next steps will surely involve an exploration of the cellular and ecological diversity of Asgards and their symbiotic partners, as well as in-depth study of the structure and activities of archaeal homologues of key eukaryotic proteins in vitro*,* and their potential functions in a few experimentally tractable model systems, like *Sulfolobus*. The success in culturing *P. syntrophicum* from environmental samples also makes clear how much there is yet to learn about microbial diversity in nature. Indeed, there is a real chance of identifying other intermediates in stable partnerships with proteobacteria that might represent sister groups that are even closer to eukaryotes than Lokiarchaeota (depicted by a “?” in Fig. 1). Thus, while we now know so much more about the origin of eukaryotes than we did in 2014, fleshing out the details remains a thrilling prospect for the years ahead.

## References

[1] Williams TA, Foster PG, Cox CJ, Embley TM (2013). An archaeal origin of eukaryotes supports only two primary domains of life. Nature.

[2] Williams TA, Cox CJ, Foster PG, Szöllősi GJ, Embley TM (2020). Phylogenomics provides robust support for a two-domains tree of life. Nature Ecol Evol.

[3] Baum DA, Baum B (2014). An inside-out origin for the eukaryotic cell. BMC Biol.

[4] Lindås AC, Bernander R (2013). The cell cycle of archaea. Nat Rev Microbiol.

[5] Lindås AC, Karlsson EA, Lindgren MT, Ettema TJ, Bernander R (2008). A unique cell division machinery in the Archaea. Proc Natl Acad Sci U S A.

[6] Samson RY, Obita T, Freund SM, Williams RL, Bell SD. A role for the ESCRT system in cell division in Archaea. Science. 2008;322:1710–3. 10.1126/science.1165322 PMC4121953 19008417

[7] Ellen AF, Albers SV, Huibers W, Pitcher A, Hobel CFV. Proteomic analysis of secreted membrane vesicles of archaeal *Sulfolobus* species reveals the presence of endosome sorting complex components. Extremophiles. 2009;13:67. 10.1007/s00792-008-0199-x 18972064

[8] Spang A, Saw JH, Jørgensen SL, Zaremba-Niedzwiedzka K, Martijn J, Lind AE, van Eijk R, Schleper C, Guy L, Ettema TJG (2015). Complex archaea that bridge the gap between prokaryotes and eukaryotes. Nature.

[9] Imachi H, Nobu MK, Nakahara N, Morono Y (2020). Isolation of an archaeon at the prokaryote–eukaryote interface. Nature.

[10] Low HH, Löwe J (2006). A bacterial dynamin-like protein. Nature.

